# Measuring Temporal Differences in Rural Canadian Children’s Moderate-to-Vigorous Physical Activity

**DOI:** 10.3390/ijerph17238734

**Published:** 2020-11-24

**Authors:** Brenton L. G. Button, Andrew F. Clark, Gina Martin, Megan Graat, Jason A. Gilliland

**Affiliations:** 1 Human Environments Analysis Laboratory, Western University, London, ON N6A 3K7, Canada; aclark2@uwo.ca (A.F.C.); gmarti57@uwo.ca (G.M.); mgraat@uwo.ca (M.G.); jgillila@uwo.ca (J.A.G.); 2 Department of Geography and Environment, Western University, London, ON N6A 5C2, Canada; 3 Children’s Health Research Institute, London, ON N6A 5A5, Canada; 4 Department of Epidemiology & Biostatistics, Western University, London, ON N6A 5C1, Canada; 5 School of Health Studies, Western University, London, ON N6A 3K7, Canada; 6 Department of Paediatrics, Western University, London, ON N6A 3K7, Canada; 7 Lawson Health Research Institute, London, ON N6C 2R5, Canada

**Keywords:** rural population, physical activity, children, temporal

## Abstract

The purpose of this study was to measure the factors that influence children’s moderate-to-vigorous physical activity (MVPA) during school curriculum time, recess time, and outside school time in a rural area. During the Fall and Winter of 2016, 34 boys and 55 girls aged 8–14 years from rural communities in rural Northwestern Ontario participated in the Spatial Temporal Environment and Activity Monitoring project. The children’s MVPA was measured using an accelerometer, and child-level demographic, behavioral, and environmental data were gathered from surveys, passively logging global positioning units, and municipal datasets. Data on daily temperature and precipitation were gathered from the closest Environment Canada weather station. A mixed model was used to assess the relationship between child- and day-level factors and children’s MVPA. On average, children were getting 12.9 min of MVPA during recess, 17.7 min during curriculum time, and 29.0 min of MVPA outside school time. During all three time points, boys were more active than girls. During curriculum time, children in lower grades were more active, and the weather had differing impacts depending on the time of day. The findings of this study illustrate the differences in MVPA and the factors that influence MVPA by time of day. Examining different time segments provides valuable information for understanding children’s MVPA patterns.

## 1. Introduction

Over the past decade, research has shown that children in industrialized countries are participating in an inadequate amount of physical activity (PA), especially at moderate-to-vigorous intensities [1,2,3]. It is imperative that moderate-to-vigorous PA (MVPA) levels increase, as studies suggest that there is a dose–response relationship between MVPA and health benefits [4]. Specifically, higher levels of MVPA have been associated with decreasing the risk of developing non-communicable diseases and lowering cardiometabolic risk factors [4].

People who live in rural areas are often shown to be a vulnerable population, with a higher prevalence of health concerns related to low levels of MVPA, such as lower functional health and higher obesity rates [5,6]. With no clear evidence regarding the extent to which MVPA levels differ between urban and rural environments, it is critical to understand the determinants of MVPA in rural regions to prevent further health inequity [7,8].

Researchers have suggested that children’s MVPA varies by day type, as children are more physically active during the week than on weekends [9]. The major difference between weekdays and weekends is children’s attendance in school. Some research has explored children’s weekday MVPA by separately examining curriculum time, recess time, and out-of-school time [10]. These different temporal periods have inherent characteristics that can interact with other variables that influence the amount of MVPA children achieve. MVPA during curriculum time is often confined to a specific area, uses certain equipment, is teacher led, and is done with peers. Higher levels of MVPA during this time have been associated with gender, single-gender classes [11], smaller classes, and access to facilities [12]. MVPA during recess is when children have greater freedom to choose their activities under the overarching school rules and policies. Although it is a well-researched subject, there is a lack of conclusive factors that influence MVPA during recess, but more facilities, unfixed equipment, and gender (i.e., boys being more active than girls) seem to be consistently related to recess-time MVPA [13]. Out of school, children have more free time and freedom to choose where they participate in PA or sedentary behaviours, either inside or outside their home neighborhood [14]. Some of the variables associated with out-of-school-time MVPA include gender (i.e., boys being more active than girls), younger age, positive motivation, time spent outside, and access to a supportive environment [15]. These three different temporal time periods are further impacted by different weather conditions, as both temperature and precipitation have been shown to influence children’s MVPA [16].

This research body on children’s MVPA offers a valuable starting point but misses the potential nuances of living in a rural area. For instance, in some rural schools, children have smaller class sizes and have health and physical education in the gymnasium every day, whereas in larger urban schools, children might have limited opportunities to use the facilities due to scheduling conflicts based on school size [17]. During recess in rural areas, children typically have larger, more naturalised playgrounds than in urban areas, which have been associated with an increase in PA [18]. Outside school hours, rural children typically have less access to recreation facilities than urban children, and researchers have demonstrated that increased access leads to higher levels of MVPA in urban children [19,20,21]. Despite these differences in opportunities for MVPA between rural and urban areas, little is known about the factors related to rural children’s MVPA during these three different time segments.

The purpose of this paper is to examine the factors that influence rural children’s MVPA during school curriculum time, school recess, and out-of-school time during weekdays in a rural area in Canada. By addressing this purpose, this paper can inform researchers, school officials, and health promoters on how to direct interventions to increase MVPA among children in rural settings on weekdays.

## 2. Materials and Methods 

### 2.1. Study Location

The common discourse on the rural environment is generally focused on agricultural communities. This approach further marginalizes children from different types of rural areas. The study communities were in rural Northern Ontario, Canada, and included children from three townships: Nipigon (population, 1642), Red Rock (population, 895), Dorion (population, 316), the dispersed rural community of Hurkett (population, 236), and Lake Helen 53A Indian Reserve (population, 303) [22]. Like many rural areas across North America, the four study communities are former resource-extraction communities. These communities are still trying to find major employers and face many issues at the forefront of rural health, including limited public health infrastructure and economic decline.

### 2.2. Study Design and Data Collection

This study used data collected as part of the Spatial Temporal Environment and Activity Monitoring (STEAM) project, which is described in other published work [21,23]. Specifically, this paper used similar methods to Button et al. [16]. In consultation with each school’s principal, ethics were submitted and approved by Western University’s Non-Medical Research Ethics Board, and the study was performed in accordance with the Declaration of Helsinki (NMREB File Number: 108029). Using a convenience sampling approach, all schools with Grades 4–8 in the study communities were invited and agreed to participate in the study. Before the children were informed about the study, each school used its social media to connect with parents to inform them about the study so they could ask questions or attend the in-school presentations. The research team gave a brief presentation to each class about the research project, and a letter of information, a parent survey, and a parental consent form were distributed to each student to take home. For a child to participate, they had to return a signed parental consent form and provide their own assent. The study was conducted over two periods (16 days in total); the first round of data collection occurred during late September and early October, and the second round began in late November and lasted to mid-December during the same school year.

Both the parent survey and the child survey asked questions about demographic characteristics, PA, health behaviours, and neighborhood perceptions. The survey questions were based on the Neighborhood Environment and Walkability Survey [24], the Paediatric Quality of Life Measurement Model (PedsQL) [25], and other highly used surveys [21]. As the children were filling out their surveys, they were individually fitted with waist-worn accelerometers and passively logging global positioning system (GPS) (Visiontac VGPS-900 or Columbus V-900) units. The children were instructed to wear the equipment during the study, including during sporting activities such as ice hockey practice, but with the exception of water-based activities. To ensure compliance with proper equipment usage and functioning, the research team visited every school each weekday.

The four regional schools had 194 students from Grades 4 to 8 (ages 8–14), of whom 134 students had parental consent and provided their own assent to participate in this study (69.1%). This sample was reduced for further analysis based on the following criteria: (a) the child was required to meet the accelerometer wear-time criteria described in the following section, (b) the child had to have at least one valid accelerometer weekday in Fall and at least one in Winter, (c) both the child and parent completed the relevant questions in the surveys, and (d) the child’s home location was identified by GPS. After applying all four inclusion criteria, a final sample of 89 children with a total of 528 valid days of data was available for further analysis (see Figure S1).

### 2.3. Dependent Variable: Moderate-to-Vigorous PA (MVPA)

The dependent variable used in this study was the minutes of MVPA during recess, curriculum time, and out-of-school time per day. The time blocks were determined by each school’s daily schedule, and time-coded accelerometer data were organised as either recess (based on bell schedules), curriculum time (from the first bell to the final bell, excluding recess), or out-of-school time (all valid data out of school time). MVPA was measured using an Actical^®^ Z Accelerometer (Philips Respironics, Murrysville, PA, USA), an omnidirectional device worn around the hips sitting on either hipbone, and the data were processed similar to in other STEAM studies [21,26]. The accelerometer was set to measure PA in 30 s epochs. This length has been used previously for this age group [27]. The accelerometer was set to record the movements made by each participant in all directions, summed over a one-minute period (counts per minute, or CPM). Each student had a minimum of at least one valid day in both seasons. Over 97.8% of the sample had three or more valid days. For invalid wear time to be considered, the accelerometer had to have zero counts for 60 consecutive minutes. For a day to be considered in this analysis, it had to have at least 10 h of wear time [28]. MVPA was considered to be at least 1500 counts per minute [29].

### 2.4. Independent Variables 

The independent variables used in this study were measured at either the day- or child-level; all the variables were selected for inclusion in the analysis based on findings from previous research that found that these variables impacted children’s PA. The child-level variables were derived from previously used surveys and were based on face validity, except for the physical environment variable, which used GPS data, and the school day variable, which used administrative data.

The day-level variables included maximum temperature and two binary variables that were used to measure precipitation: snow (snow vs. no snow) and rain (rain vs. no rain). The day-level data came from the closest Environment Canada weather station. The child-level variables from the child survey included grade (continuous) [15], gender (boy vs. girl) [15], ethnicity (Caucasian/white vs. Indigenous and visible minority) [30,31], perception of physical functioning (below or above the median based on questions from the PedsQL physical functioning scale) [32], parental support (agree vs. disagree whether a parent takes part in activities with you) [15], whether the child perceived that they lived within walking distance to the school (yes vs. no) [33], and perceptions of social, neighborhood, and safety barriers (continuous) [26]. The social, neighborhood, and safety barriers for PA were constructed using a four-point Likert scale, in which the participants responded to statements about the perceptions of barriers related to PA in their general neighborhood or areas where they played, their safety, and their social environment; the scores ranged from −2 to 2 [26]. The physical functioning measure was created using questions from the PedsQL regarding how difficult basic activities were for the child [25]. The Cronbach’s Alpha for the physical functioning variable was 0.820, which is generally considered to be acceptable [34]. The questions were scored based on the guidelines from the PedsQL and then dichotomised as below or above the median. From the parent survey, the mother’s education (high school or below vs. college or above) [35] and family composition (two-parent household vs. one-parent household) [36] were obtained. Using the children’s GPS-derived home location, a physical environment variable was created, and the children were grouped into two categories based on whether they lived in a more settled area or the more rural areas surrounding the community (rural small town vs. rural). Children who lived in settled areas typically had walking access to the community recreation centre or parks. 

Finally, at the child-level, based on the school schedule, children were categorised as attending a balanced or traditional school [37]. A balanced school day (BSD) has three academic periods (about 100 min in length) separated by two breaks (about 40 to 45 min). In a traditional school day (TSD), children have two recesses (about 15 min in length) and a lunch break (about 45–60 min). Playgrounds were examined using the Ontario Physical and Health Education Association audit tool, and no differences were found between the playgrounds, so no variables measuring playgrounds were included [38]. Only the variables of maternal education and whether a child reported they lived within walking distance had missing data, with less than 10% of cases missing. Data were imputed using a mode fill [39].

### 2.5. Statistical Analyses

A linear mixed model was fit to examine children’s daily MVPA levels during curriculum time, recess time, or out-of-school time while including child-level (e.g., age and gender) and day-level (e.g., temperature and precipitation) factors. In this study, the daily MVPA values were clustered around the child and the day the data were collected. Essentially, all MVPA data that came from one child were more consistent when compared with other children, and data that came from a specific calendar day were more alike when compared with other calendar days. Using a mixed model, researchers can account for these data structures [40]. To ensure that a mixed model was appropriate, two empty models for each of the three specific day times were tested: a date model and a child model. Both models were significant (*p* < 0.05), justifying the mixed model approach.

In the recess time and curriculum time models, a forced-entry method was used, which only considered grade, gender, ethnicity, physical functioning, a BSD or TSD, maximum temperature, and precipitation. The out-of-school time model also used a forced-entry method but included grade; gender; ethnicity; physical functioning; parental support; whether the child perceived that they lived within walking distance to the school; perceptions of social, neighborhood, and safety barriers; mother’s education; family composition; physical environment; maximum temperature; and precipitation. Different variables were tested in the out-of-school time model, as, during school, children are confined to school property, so a variable such as physical environment is not impactful during the school day but could be important during out-of-school time.

All three mixed models followed the same progression. First, a null model provided an estimate of the variance of the daily MVPA values across children and across dates. Second, the child-level variables were added to the null model to understand how they influenced MVPA. Third, the day-level variables were added to the null model to understand how they influenced the daily values of MVPA. Finally, the child-level and day-level factors were added together to provide an understanding of how the two types of factors interacted to influence daily values of MVPA during the three distinct time blocks. All data analysis was conducted in SAS 9.4 (SAS Institute Inc., Cary, NC, USA).

## 3. Results

Table 1 presents the descriptive statistics for the continuous variables and the frequencies for categorical variables included in the analysis. On average, children were getting 12.9 min of MVPA during recess, 17.7 min during curriculum time, and 29.0 min of MVPA during out-of-school time. There was an even distribution from Grades 4 to 7, with each grade contributing about 20 children, but in Grade 8, only 10 children participated. There were more girls (62%) than boys (38%), and 58% of children reported being Caucasian/white, while 42% reported Indigenous or other. The average maximum temperature was 8.9 °C (48.0 °F).

### 3.1. Recess

In Table 2, Model 1 contains all the child-level characteristics. This analysis found that only gender was significant. On average, boys were getting 6.38 more minutes of daily MVPA (b = 6.38; *p* < 0.01) when compared with girls. Model 2 represents the addition of three day-level variables. On average, for each 1 °C increase in maximum daily temperature, children were getting 14 more seconds of MVPA per day (b = 0.24; *p* < 0.01). In Model 3, both child- and day-level variables were represented. Boys were significantly more active than girls, getting, on average, 6.38 more minutes of MVPA (b = 6.38; *p* < 0.01). Finally, for each 1 °C increase in maximum daily temperature, children were getting 14 more seconds of daily MVPA, on average (b = 0.24; *p* < 0.01).

### 3.2. Curriculum

In Table 3, Model 1 contains all the child-level characteristics. This analysis found that gender and grade were significant. On average, boys were getting 6.47 more minutes of MVPA a day (b = 6.47; *p* < 0.01) than girls, and as the children increased in grade level, the children were getting 2.32 fewer minutes of MVPA per day (b = −2.32; *p* < 0.01). In Table 3, Model 2 represents the addition of three day-level variables, and, when comparing days with rain to days without rain or snow, children were getting 9.24 fewer minutes of MVPA (b = −9.24; *p* = 0.03). In Table 3, Model 3 contains both child- and day-level variables. Boys were significantly more active than girls, getting, on average, 6.55 more minutes of MVPA per day (b = 6.55; *p* < 0.01). Each increase in grade was related to a 2.33 min decrease in daily MVPA (b = −2.33; *p* < 0.01), and comparing days with rain to days without rain, children were getting 10.50 fewer minutes of MVPA per day during days with rain (b = −10.50; *p =* 0.01).

### 3.3. Out of School

In Table 4, Model 1 contains all the child-level variables. This analysis found that only gender was significant. On average, boys were getting 15.96 more minutes of MVPA than girls out of school (b = 15.96; *p* < 0.01). Model 2 represents the addition of three day-level variables. For each 1 °C increase in daily maximum temperature, children were getting 36 more seconds of MVPA per day (b = 0.60; *p* < 0.01). In Table 4, Model 3 contains both child- and day-level variables. Boys were significantly more active than girls, with an average of 16.01 more minutes of MVPA per day out of school (b = 16.01; *p* < 0.01). Finally, for each 1 °C increase in daily maximum temperature, children were getting 36 more seconds of MVPA per day (b = 0.60; *p* < 0.01).

## 4. Discussion

This paper examined the factors that influence children’s MVPA during recess, curriculum time, and out-of-school time in rural areas. Research has shown that PA levels differ during these periods, and this paper contributes to the literature by identifying the specific factors that influence children’s MVPA during these specific times in the rural environment. The findings indicate that, on average, children were getting 29.0 min of MVPA out of school, 12.9 min during recess, and almost 18 min during curriculum time. During all the time points, boys were more active than girls, and grade was a significant predictor of MVPA during curriculum time, but not during recess or out-of-school time. Weather had varying impacts depending on the part of the day, as temperature impacted MVPA out of school and during recess time, while rain influenced curriculum time MVPA. Understanding these subtle differences allows researchers to create more tailored interventions.

This study found that, on average, boys were more active than girls, and numerous studies have discussed the differences between boys’ and girls’ PA [41,42]. This study found a unique time-segmented gender gap, with a six-minute difference in MVPA in favour of boys during curriculum time and recess time, and a sixteen-minute difference during out-of-school time. This finding suggests there is a complex relationship between space, time, and gender. To improve girls’ MVPA, these different temporal segments need to be considered, as they could relate to different intervention strategies. For example, in rural schools, children rarely have access to physical education specialists. Adding a professional development module to increase girls’ MVPA during curriculum time could be helpful, as research has suggested that generalist teachers report barriers to teaching gym class [43,44]. Out of school, some rural-specific research suggests that gender-separated activities could be beneficial, as girls and boys enjoy different activities [45]. However, in this rural region, gender-specific activities have been attempted but are difficult to sustain. Future research is needed on specific rural interventions for girls.

During curriculum time, children were only getting an average of 18 min of MVPA, and there was about a two-minute decrease in MVPA as children increased in grade level. In this particular area, a daily PA policy of 20 min of MVPA exists, but the majority of children are not reaching this level [46]. A potential explanation from Dwyer et al. (2003) for this failure is that physical education is considered a low-priority subject, and as children advance from grade to grade, the teachers focus more on numeracy and literacy [47]. Without meaningful assessment or evaluation of the program, it will continue to fail to meet the needs of students. It is imperative that research continues to address the potential shortcomings of this policy, specifically in rural areas, as rural schools offer children opportunities to be active with friends in a supportive environment [48].

In contrast to previous literature, this study found no difference in MVPA during recess or out-of-school time across grade levels [49]. This lack of difference could stem from living in a rural environment. To play larger team-based games at recess and out of school, there needs to be a mix of both younger and older children to make complete teams. A further understanding of the dynamics across age in rural areas is an important research topic, as rural areas typically offer fewer programs than urban areas [50]. If researchers can create educational programs for coaches, recreation programmers, and volunteers in rural communities that are focused on creating appropriate activities for groups of children at different developmental stages, this could help to increase the sustainability of recreation programs.

Similar to other weather and seasonal studies, rural children were found to be more active in warmer weather during out-of-school and recess time, and rain only impacted curriculum time [51]. Some research has claimed that a decrease in MVPA between seasons is related to children’s time spent outdoors [52]. In the current study area, children are expected to go outside for recess at all times of the year, but there was still a decrease in MVPA during colder temperatures, suggesting that the difference in MVPA is not entirely explained by indoor and outdoor time. School administrators need to find ways to boost recess MVPA as the temperature drops. Out-of-school time rural research has often indicated that a lack of indoor facilities is a potential barrier to MVPA, specifically in colder weather [16,53]. To overcome this barrier, researchers have suggested creating more indoor programs to increase children’s MVPA. However, it is unclear whether this intervention would work, as rural children still face the barriers of transportation, limited people to play with, and limited program offerings. Rural researchers must develop an in-depth understanding of all the facilitators and barriers and how they potentially interact in specific communities.

This research also had some null findings, which were noteworthy, given that they differ from findings in previous studies. Previous research, which has mostly focused on children in urban settings, found that factors such as parental support and active transportation have an influence on MVPA [48,54,55]. Researchers have suggested that families can play an important role in a child’s MVPA [55]. In this paper, children’s perceptions of parental support and whether a child came from a one- or two-parent household had no influence on MVPA. A potential explanation for these findings is that some parents who live in remote or low-density rural communities, such as those in Northern Ontario, have to leave their home community for employment and spend extended periods away from home (i.e., 21 days at work and 7 days at home). In these situations, there might only be one parent in the household, making it difficult to support their child’s afterschool PA. Another interesting null finding is that living within walking distance of school did not have a statistical impact on children’s MVPA. Active transportation has been cited as an important contributor to MVPA [54]. In rural areas, children are less likely to use active modes of transportation [56]. Researchers have suggested that the infrastructure in rural communities typically does not support active transportation, as many of these communities do not have adequate sidewalks or bike lanes [56,57]. Additionally, in low-density rural communities, people are car-dependent and travelling by car is seen as the norm for most trips [56,58]. A potential intervention could aim to increase resident knowledge on the importance of active transportation, while decreasing built environment barriers [59]. Finally, rain had a significant impact on MVPA during curriculum time, but not during recess or outside school time. During curriculum time on nice days, teachers might be more willing to extend recess for a few minutes or go outside before the bell rings and give children an opportunity to play, whereas they might be less likely to do this on rainy days. In afterschool time, children are potentially at home playing inside regardless of the weather.

A potential limitation of this study is how the “school day” was defined. In this study, the school day was based on the first bell to the final bell. This definition does not include the time when children arrive at school early, as this time could be similar to recess, given that children are confined to the school ground and subject to school rules. However, it is difficult to determine when children arrived at school and whether they were subject to regular school playground rules in that pre-school time. Another consideration is that the time available for PA differs between recess, curriculum, and out-of-school time, so a larger difference between boys’ and girls’ MVPA during out-of-school time is logical, as there is more time available to be active. Finally, since this study only examined quantitative measures of children’s MVPA, it is difficult to make specific activity-based program recommendations.

## 5. Conclusions

Rural areas seem to be at a continuous disadvantage compared with urban areas. A lack of data and rigorous studies has been cited as a significant research gap for rural areas [48]. Using accelerometers, this study illustrated how different factors are related to MVPA during different parts of the weekday among rural children. To reduce the gap between boys’ and girls’ MVPA, communities and schools need to work with girls to determine the barriers that prevent them from being more active during all parts of the weekday. During curriculum time, teachers of older students need to find ways to motivate the children and to create lesson plans that keep children moving, so that students reach the MVPA threshold. Finally, during recess and out-of-school time throughout the Winter, rural children need more accessible indoor opportunities [16,60]. Ultimately, breaking down a child’s day into different time points allows for more specific and potentially successful interventions.

## Figures and Tables

**Table 1:** Study characteristics for the 528 days of data from 89 children.

Dependent Variable	Mean and SD
MVPA during recess	12.9 (9.7)
MVPA during curriculum time	17.7 (13.6)
MVPA out of school	29.0 (25.5)
Child-Level	Count and %
Gender	
Boys	34 (38.2)
Girls	55 (61.8)
Ethnicity	
Caucasian	52 (58.4)
Indigenous or visible minority	37 (41.6)
Grade	
Four	18 (20.2)
Five	20 (22.5)
Six	20 (22.5)
Seven	21 (23.6)
Eight	10 (11.2)
Parental support	
Agree	50 (56.2)
Disagree	39 (43.8)
Mother’s education	
High school and below	21 (23.6)
College and above	68 (76.4)
Family composition	
One-parent household	13 (14.6)
Two-parent household	76 (85.4)
Live within walking distance to school	
Yes	56 (62.9)
No	33 (37.1)
Social barrier *mean* (*sd*)	−0.6 (0.7)
Neighborhood barrier *mean* (*sd*)	−0.8 (0.6)
Safety barrier *mean* (*sd*)	−1.2 (0.8)
Physical functioning *mean* (*sd*)	88.8 (15.9)
Physical environment	
Rural	46 (51.7)
Rural small town	43 (48.3)
School day	
Balanced	67 (75.3)
Traditional	22 (24.7)
Day-Level	Count and %
No rain or snow	11 (57.9)
Cases of rain Cases of snow Maximum temperature °C mean (*sd*)	3 (15.8) 5 (26.3) 8.9 (9.5)

**Table 2:** The mixed model assessing the relationship between children’s recess MVPA and child variables (Model 1), day variables (Model 2), and child- and day-level variables (Model 3).

Variable	Category	Model 1			Model 2			Model 3		
		Est	SE	*p*-value	Est	SE	*p*-value	Est	SE	*p*-value
Intercept		13.80	3.45		10.22	1.35		11.84	3.56	
Gender (*ref: Girls*)	Boys	6.38	1.40	**<0.01**				6.38	1.40	**<0.01**
Grade		−0.59	0.51	0.25				−0.61	0.51	0.23
Ethnicity (*ref: Caucasian*)	Indigenous or visible minority	0.43	1.38	0.75				0.53	1.38	0.70
Physical functioning *(ref: Low*)	High	0.08	1.45	0.97				0.02	1.46	0.99
School day (*ref: BSD*)	TSD	−1.84	1.73	0.29				−2.17	1.67	0.19
Maximum temperature					0.24	0.07	**<0.01**	0.24	0.07	**<0.01**
Rain days (*ref: No*)					−0.58	1.96	0.77	−0.91	1.92	0.63
Snow days (*ref: No*)					0.95	1.58	0.55	0.79	1.53	0.60

Italics indicate reference group. Values in boldface indicate statistical significance; *p*-value < 0.05.

**Table 3:** The mixed model assessing the relationship between children’s curriculum time MVPA and child variables (Model 1), day variables (Model 2), and child- and day-level variables (Model 3).

Variable	Category	Model 1			Model 2			Model 3		
		Est	SE	*p*-value	Est	SE	*p*-value	Est	SE	*p*-value
Intercept		27.46	3.89		17.19	2.73		28.03	4.47	
Gender (*ref: Girls*)	Boys	6.47	1.47	**<0.01**				6.55	1.46	**<0.01**
Grade		−2.32	0.53	**<0.01**				−2.33	0.53	**<0.01**
Ethnicity (*ref: Caucasian*)	Indigenous or visible minority	−0.82	1.46	0.58				−0.69	1.45	0.63
Physical functioning (*ref: Low)*	High	1.68	1.52	0.27				1.76	1.51	0.25
School day *(ref: BSD)*	TSD	−1.37	1.97	0.48				−1.70	1.94	0.38
Maximum temperature					0.15	0.17	0.38	0.16	0.18	0.36
Rain days *(ref: No)*					−9.24	4.18	**0.03**	−10.50	4.29	**0.01**
Snow days *(ref: No)*					−1.83	3.56	0.61	−2.16	3.65	0.55

Italics indicate reference group. Values in boldface indicate statistical significance; *p*-value < 0.05.

**Table 4:** The mixed model assessing the relationship between children’s out of school MVPA and child variables (Model 1), day variables (Model 2), and child- and day-level variables (Model 3).

Variable	Category	Model 1			Model 2			Model 3		
		Est	SE	*p*-value	Est	SE	*p*-value	Est	SE	*p*-value
Intercept		24.49	9.01		22.40	3.36		19.72	9.00	
Gender *(ref: Girls)*	Boys	15.96	3.35	**<0.01**				16.01	3.34	**<0.01**
Ethnicity (*ref: Caucasian)*	Indigenous or visible minority	−1.24	3.52	0.73				−1.37	3.51	0.70
Grade		0.27	1.22	0.83				0.24	1.23	0.84
Physical functioning *(ref: Low)*		2.13	3.48	0.54				1.66	3.47	0.63
Parental support *(ref: Disagree)*	Agree	−1.59	3.41	0.64				−1.56	3.40	0.65
Maternal education *(ref: High School or Below*)	College or above	−3.86	2.96	0.19				−3.96	2.94	0.18
Family composition (*ref: Two)*	One	0.38	4.68	0.94				0.77	4.67	0.87
Live within walking distance to school *(ref: No)*	Yes	5.24	3.20	0.10				4.95	3.18	0.12
Social barrier		−0.71	2.46	0.77				−0.73	2.46	0.77
Neighborhood barrier		0.46	2.65	0.86				0.31	2.63	0.91
Safety barrier		2.34	2.16	0.28				2.28	2.16	0.29
Physical environment (*ref: Rural small town*)	Rural	−2.37	4.86	0.63				−2.61	4.25	0.54
Rain days *(ref: No)*					−2.00	5.54	0.72	−3.01	5.67	0.59
Snow days (*ref: No*)					2.08	3.48	0.55	1.68	3.51	0.63
Maximum temperature					0.60	0.19	**<0.01**	0.60	0.19	**<0.01**

Italics indicate reference group. Values in boldface indicate statistical significance; *p*-value < 0.05.

## Supplementary Materials

The following are available online at https://www.mdpi.com/1660-4601/17/23/8734/s1, Figure S1: FLOW diagram of final sample size.
