# Supplementary figures and images for: Measuring Temporal Differences in Rural Canadian Children’s Moderate-to-Vigorous Physical Activity
Source: Int J Environ Res Public Health. 2020 Nov 24;17(23):8734. doi: 10.3390/ijerph17238734 (PMC7727807; doi:10.3390/ijerph17238734)

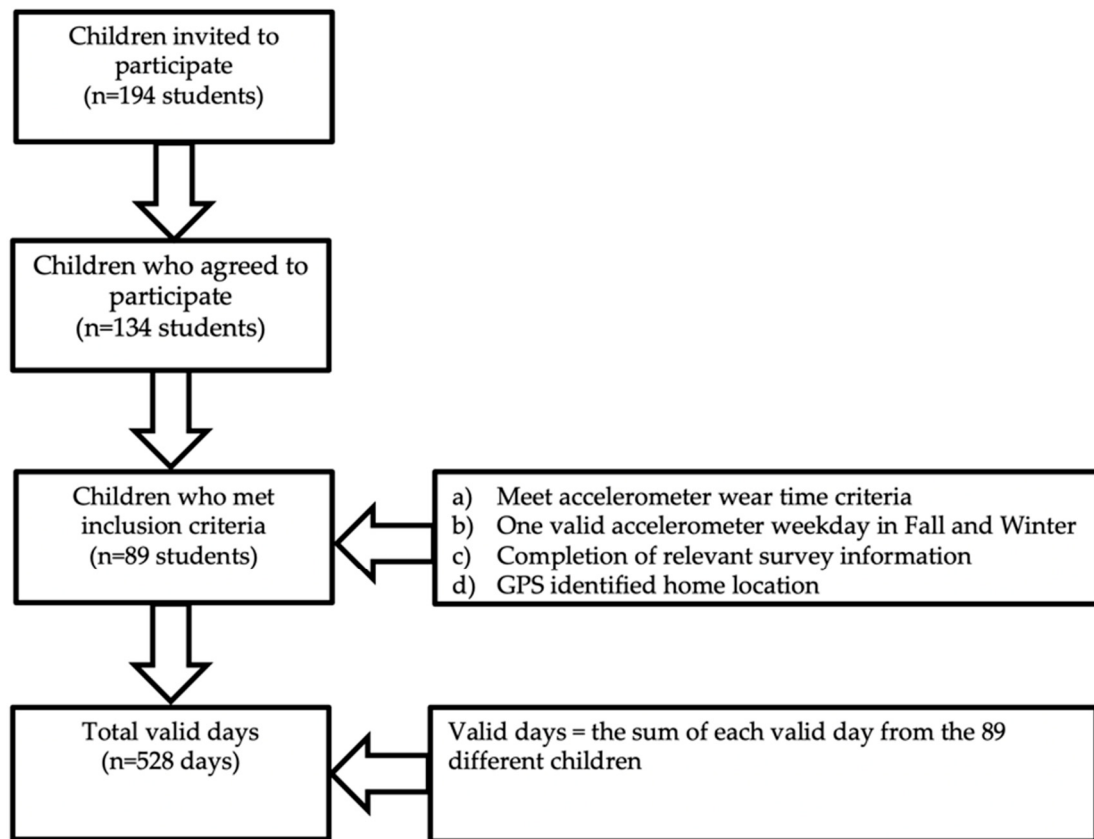

Figure S1. FLOW diagram of the final sample size.

Supplement: Supplementary file 1 [file ijerph-17-08734-s001.pdf]
